# Factors Involved in Removing the Non-Structural Protein of Foot-and-Mouth Disease Virus by Chloroform and Scale-Up Production of High-Purity Vaccine Antigens

**DOI:** 10.3390/vaccines10071018

**Published:** 2022-06-24

**Authors:** Sun Young Park, Sim-In Lee, Jong Sook Jin, Eun-Sol Kim, Jae Young Kim, Ah-Young Kim, Sang Hyun Park, Jung-Won Park, Soonyong Park, Eun Gyo Lee, Jong-Hyeon Park, Young-Joon Ko, Choi-Kyu Park

**Affiliations:** 1 Center for FMD Vaccine Research, Animal and Plant Quarantine Agency, 177 Hyeoksin-8-ro, Gimcheon-si 39660, Korea; sun3730@korea.kr (S.Y.P.); lunark321@gmail.com (S.-I.L.); in75724@korea.kr (J.S.J.); kesol13@hanmail.net (E.-S.K.); ivorikim@korea.kr (J.Y.K.); mochsha@korea.kr (A.-Y.K.); shpark0205@korea.kr (S.H.P.); parkjw6254@korea.kr (J.-W.P.); parkjhvet@korea.kr (J.-H.P.); 2 Animal Disease Intervention Center, College of Veterinary Medicine, Kyungpook National University, Daegu 41566, Korea; 3 Bioprocess Engineering Center, Korea Research Institute of Bioscience and Biotechnology (KRIBB), 30 Yeongudanjiro Ochang-eup, Chungju-si 28116, Korea; sypark@kribb.re.kr (S.P.); eglee@kribb.re.kr (E.G.L.)

**Keywords:** foot-and-mouth disease virus, non-structural protein, vaccine purity, chloroform, scale-up

## Abstract

Foot-and-mouth disease (FMD) is an economically important and highly infectious viral disease, predominantly controlled by vaccination. The removal of non-structural proteins (NSPs) is very important in the process of FMD vaccine production, because vaccinated and naturally infected animals can be distinguished by the presence of NSP antibodies in the FMD serological surveillance. A previous study reported that 3AB protein, a representative of NSPs, was removed by chloroform treatment. Therefore, in this study, the causes of 3AB removal and factors affecting the effect of chloroform were investigated. As a result, the effectiveness of chloroform differed depending on the virus production medium and was eliminated by detergents. In addition, it was found that 3AB protein removal by chloroform is due to the transmembrane domain of the N-terminal region (59–76 amino acid domain). Further, industrial applicability was verified by applying the chloroform treatment process to scale-up FMD vaccine antigen production. A novel downstream process using ultrafiltration instead of polyethylene glycol precipitation for high-purity FMD vaccine antigen production was established. This result will contribute toward simplifying the conventional process of manufacturing FMD vaccine antigens and ultimately reducing the time and cost of vaccine production.

## 1. Introduction

Foot-and-mouth disease (FMD) is an economically important and highly infectious viral disease in most parts of the world that is predominantly controlled by immunization strategies [1]. The etiological agent, FMD virus (FMDV), belongs to the genus *Aphthovirus* within the family *Picornaviridae* and is divided into seven serotypes (O, A, Asia1, South African Territories 1, 2, 3, and C), with no cross-protection between serotypes [2,3]. It has a positive-sense, single-stranded RNA genome that is translated into a polyprotein that is further cleaved into structural proteins (SPs) and non-structural proteins (NSPs) [4,5].

The removal of NSPs is an important process for FMD vaccine production, because vaccinated and naturally infected animals can be distinguished by the presence of NSP antibodies in FMD serological surveillance [6]. In this regard, polyethylene glycol (PEG) precipitation is usually used in the downstream processing of FMD vaccine production. PEG precipitation of FMDV antigens is often carried out after ultrafiltration and is predominantly used for concentration and partial purification of FMDV from various contaminants, including NSPs in FMDV culture supernatants [7,8]. PEG treatment also facilitates buffer changes. PEG is a nontoxic, water-soluble synthetic polymer that is widely used in chemical and biomedical industries. The basic mechanism of precipitation involves changing the solubility of the solvent by adding reagents to lower the solubility of the solute [9]. However, since this is a non-specific technique, NSPs could not be completely removed; therefore, some NSPs remain in the final FMD vaccine product [10,11].

Therefore, in our previous study, we established an NSP-specific removal method in which the 3AB protein was eliminated by chloroform treatment at a concentration >2% (*v*/*v*) in FMDV culture supernatants [12]. The 3AB protein was selected as a marker for NSP detection because the World Organization for Animal Health terrestrial manual specifies that 3AB and 3ABC proteins in serological analysis are the most reliable indicators of FMDV infection [13]. In addition, 3AB is a major precursor protein containing 3B in FMDV-infected cells [14]. Although the role of 3AB is unclear, it is presumed to anchor 3B to the intracellular membrane induced during the initiation of RNA replication, where uridylylated 3B primes the synthesis of nascent viral RNAs [15].

In this study, we identified the factors involved in the removal of 3AB using chloroform and established an effective and simplified process for manufacturing high-purity FMD vaccine antigens by applying the chloroform treatment process to scale-up antigen production.

## 2. Materials and Methods

### 2.1. Cells and Viruses

Baby hamster kidney-21 (BHK-21) suspension cells [16] adapted to Cellvento BHK-200 (Cellvento) (Merck KGgA, Darmstadt, Germany), ProVero-1 (ProVero) (Lonza, Basel, Switzerland), and CD BHK-200 (CD) (Thermo Fisher Scientific, Waltham, MA, USA) media were proliferated at 37 °C with 5% CO_2_ using Erlenmeyer flasks (Corning, Corning, NY, USA). The Korean isolates FMDV O/Boeun/SKR/2017 (GenBank accession No. MG983730.1), O/Jincheon/SKR/2014 (GenBank accession no. 162590.1), O/Andong/SKR/2010 (GenBank accession no. KC503937) and A/Yeoncheon/SKR/2017 (GenBank accession No. KY766148.1), foreign-origin A22 Iraq/24/64 (GenBank accession No. AY593764.1), and Asia1 Shamir/ISR/1989 were produced in each of the media described above. BHK-21 cells at a density of 3 × 10^5^ cells/mL were grown for 3.5 days, and then, the spent media were replaced/added with fresh medium (replacement: ProVero, addition: Cellvento, 30% (*v*/*v*) of total volume). Exceptionally, CD medium was replaced with Dulbecco’s modified Eagle’s medium (DMEM) (Corning). All medium combinations were optimized as described in our previous study [17]. The cells were inoculated with FMDV at a multiplicity of infection of 0.001, and virus was propagated for 16 h at 37 °C with 5% CO_2_. After culturing, the virus culture supernatant was clarified by centrifugation at 3000*× g* for 20 min at 4 °C. Binary ethylenimine (Sigma-Aldrich, St. Louis, MO, USA) was added at 3 mM to inactivate the virus and then incubated with shaking at 100 rpm at 26 °C for 28 h. Subsequently, the binary ethyleneimine was neutralized by adding 1 M sodium thiosulfate (Daejung Chemicals & Metals, Siheung, Korea) at a final concentration of 2% (*v*/*v*).

### 2.2. Expression and Purification of Recombinant 3AB Protein

The FMDV 3AB gene (O/Boeun/SKR/2017) was synthesized by Bioneer Corp. (Daejeon, Korea). The 3AB DNA fragments, (i) full-length (672 bp) and (ii) truncated (432 bp) were amplified and cloned into the pET28a vector. The recombinant plasmids were transformed into *Escherichia coli* (*E. coli*) BL21 (DE3) strain, and cells were cultured in Luria-Bertani medium containing 50 μg/mL kanamycin at 37 °C until the optical density at 600 nm reached 0.6. The expression of recombinant proteins was induced by adding 1 mM isopropyl-β-d-1-thiogalactopyranoside at 16 °C for 4 h. Pellets were harvested from the culture by centrifugation at 3000*× g* for 10 min, resuspended in lysis buffer [50 mM Na_2_HPO_4_, 300 mM NaCl, pH 8.0], and lysozyme was added to a final concentration of 1 mg/mL. After incubation on ice for 30 min, the cells were lysed by sonication (20 cycles of pulsed on-time 5 s and off-time 10 s at 20% amplitude). Cell lysates were centrifuged at 10,000*×* *g*, 4 °C for 30 min, and supernatants were mixed with nickel-nitrilotriacetic acid resin (Qiagen, Germantown, MD, USA) by inverting at 4 °C for 1 h. The mixture was loaded onto an Econo-Pac column (Bio-Rad, Hercules, CA, USA) and passed through gravity flow. The column was washed twice with wash buffer [50 mM Na_2_HPO_4_, 300 mM NaCl, and 10 mM imidazole, pH 8.0], and the recombinant 3AB protein was then eluted with elution buffer [50 mM Na_2_HPO_4_, 300 mM NaCl, and 250 mM imidazole, pH 8.0]. The eluted fractions were pooled and dialyzed against dialysis buffer [50 mM Tris-HCl and 150 mM NaCl, pH 7.6] using a Slide-A-Lyzer membrane cassette (Thermo Fisher Scientific) with a molecular weight cut-off of 10 kDa.

### 2.3. Prediction of Transmembrane Domain for 3AB

The 3AB protein sequence was analyzed using the TMHMM Server version 2.0. Based on the FASTA-formatted protein sequence, the positions of the transmembrane helices and intracellular and extracellular regions were predicted using hidden Markov models [18]. The server is available at http://www.cbs.dtu.dk/services/TMHMM/ (accessed on 5 July 2021).

### 2.4. Scale-Up Production of FMD Vaccine Antigens

A wave-mixing bioreactor (BIOSTAT Cultibag RM 20/50; Sartorius Stedim Biotech, Gottingen, Germany) was used to produce 20 L of the FMDV O/Boeun/SKR/2017 culture supernatant. BHK-21 suspension cells were grown in Cellvento medium using a single-use bag (Flexsafe RM 50 L Optical; Sartorius Stedim Biotech) by rocking motion agitation. The FMDV O/Boeun/SKR/2017 virus was inoculated at a multiplicity of infection of 0.001, and virus was propagated for 16 h at 37 °C with 5% CO_2_. The bioreactor was equipped with probes to measure and control the temperature and dissolved oxygen concentration at 37 °C and 50% air saturation, respectively. The pH was controlled in the range of 7.2–7.4 by adding CO_2_ and 1 M NaOH solution. The virus culture supernatant was collected at 16 h post-infection and clarified by centrifugation at 3000*× g* for 20 min at 4 °C. The virus was inactivated by binary ethyleneimine in a wave-mixing bioreactor using a single-use bag (Flexsafe RM 50 L Optical; Sartorius Stedim Biotech). The detailed conditions for inactivation are the same as in Section 2.1.

### 2.5. Chloroform Treatment

After virus inactivation, the virus culture supernatant was treated with chloroform. Small-scale chloroform treatment using a centrifugal tube was established in our previous study [12]. To examine the effect of chloroform treatment according to the type of virus production medium or buffer, an Amicon Ultra-15 (nominal molecular weight limit of 10 kDa) centrifugal filter unit (Merck KGgA) was used to change the various solutions. After repeating the washing process by centrifugation at 2000*× g* at 4 °C until the medium was sufficiently replaced with the buffer solution, chloroform was added to the solution and mixed by the inverting method. The virus culture supernatant in the 20 L scale was mixed with chloroform at 350 rpm for 30 min in a STD FLEXEL tank liner bag (Sartorius Stedim Biotech) with an impeller (Thermo Scientific, Catalog number: SH30749.12) connected to a single-use modular mixing system (HyPerforma DS 300; Thermo Scientific).

### 2.6. Micro and Ultrafiltration

Chloroform-treated virus culture supernatant was clarified by depth filter (nominal micron rating 0.2–2 μm) of 0.054 m^2^ Milistak plus POD C0HC (Millipore, Billerica, MA, USA) and sterile filter (0.22 micron) of 0.059 m^2^ Opticap XL 600 Express SHC (Millipore). The clarified virus supernatant was concentrated 10-fold using a Sartoflow Advanced benchtop cross-filtration system (Sartorius Stedim Biotech) equipped with a cut-off size of 300 kDa polyethersulfone Sartocon Slice membrane cassette (Sartorius Stedim Biotech). The virus culture supernatant was concentrated at 10 psi transmembrane pressure according to the manufacturer’s instructions.

### 2.7. Western Blot Analysis

The protein samples were mixed with 4× lithium dodecyl sulfate sample buffer (Invitrogen, Carlsbad, CA, USA) and heated at 90 °C for 10 min. Samples were run on 4–12% gradient bis–tris gels (Invitrogen) and then transferred to a nitrocellulose membrane using the iBlot2 gel-transfer device (Invitrogen). The membranes were blocked in buffer [PBST; 10 mM sodium phosphate, 132 mM NaCl, 2.7 mM KCl, and 0.05% Tween-20, pH 7.4] for 1 h at 25 °C with shaking, washed three times with PBS-T for 10 min, and then incubated with a home-made primary antibody against FMDV VP1 (76.5E) and 3B (4G24) diluted 1/2000 in PBST at 4 °C overnight. The membranes were washed three times with PBS-T and incubated with HRP-conjugated goat anti-mouse secondary antibody (Invitrogen) diluted 1/4000 in PBST for 1 h at RT. Proteins were detected with Pierce ECL Substrate (Invitrogen) using an Azure C600 imaging system and cSeries Capture Software (Azure Biosystems, Dublin, CA, USA).

### 2.8. Transmission Electron Microscopy (TEM)

The concentrated vaccine antigen was layered on top of the sucrose density gradient and then centrifuged at 100,000*× g* for 4 h. The fractions between the 30% and 35% sucrose layers were collected and centrifuged at 100,000*× g* for 4 h. After removing the sucrose solution, the pellet was resuspended in Tris-KCl buffer [20 mM Tris-HCl and 300 mM KCl, pH 7.6]. One drop of purified FMDV suspension was placed on formvar-coated grids and negatively stained with 1% uranyl acetate. The morphology of the purified FMDV particles was examined using TEM (Hitachi 7100; Hitachi, Tokyo, Japan).

## 3. Results

### 3.1. Comparison of Virus Production Medium and Buffer for the Effect of Chloroform to Remove 3AB

**Figure 1:**
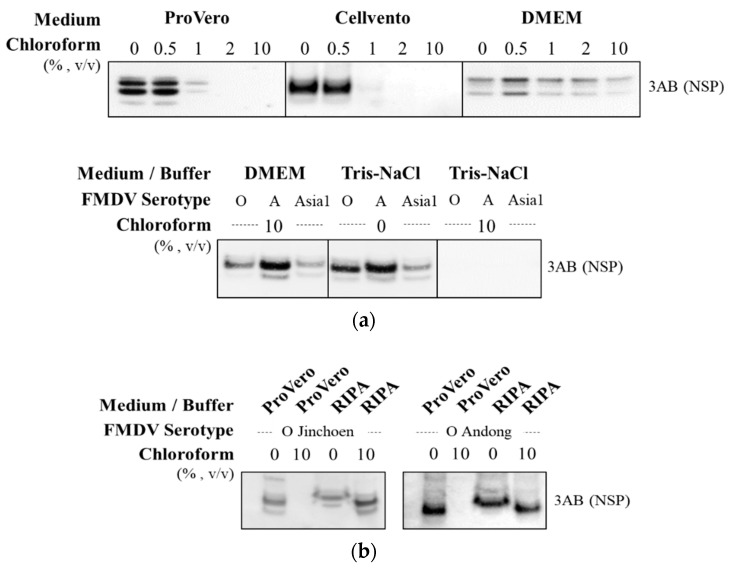
Effect of chloroform treatment according to the type of medium or buffer. (**a**) The O/Boeun/SKR/2017 virus produced in different types of cell culture medium were treated with various concentrations of chloroform (upper panel). The O/Jincheon/SKR/2014, A_22_ Iraq/24/64, and Asia1 Shamir/ISR/1989 virus were produced in Dulbecco’s modified Eagle’s medium (DMEM) and then exchanged with Tris-NaCl buffer. DMEM and Tris-NaCl buffer were each treated with 10% (*v*/*v*) chloroform (lower panel). (**b**) The O/Jincheon/SKR/2014 and O/Andong/SKR/2010 were produced in ProVero medium and then exchanged with radioimmunoprecipitation assay (RIPA) buffer. ProVero and RIPA buffer were each treated with 10% (*v*/*v*) of chloroform. The 3AB protein was detected by Western blot using anti-FMDV 3B monoclonal antibody.

We examined whether there was a difference in the effect of chloroform on the removal of NSP depending on the type of virus production medium and buffer. In the ProVero and Cellvento media, the 3AB protein was removed by chloroform treatment at a concentration of 2% (*v*/*v*) or more. However, no effect of chloroform was observed in DMEM (Figure 1a). Although there was no effect of chloroform on all three different serotypes of FMDV culture supernatants cultured in DMEM, NSPs were removed by chloroform (10%, *v*/*v*) after the medium was replaced with Tris-NaCl buffer [50 mM Tris-HCl, pH 7.6, 150 mM NaCl]. In contrast, NSP was not removed when the ProVero medium was replaced with radioimmunoprecipitation assay (RIPA) buffer [25 mM Tris-HCl, 150 mM NaCl, 1% NP-40, 1% sodium deoxycholate, 0.1% sodium dodecyl sulfate, pH 7.6] (Figure 1b).

### 3.2. Effect of Chloroform According to the Presence or Absence of 3AB Transmembrane Domain

**Figure 2:**
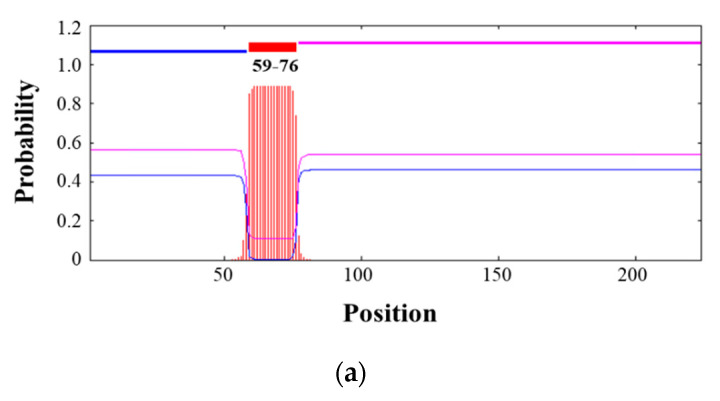

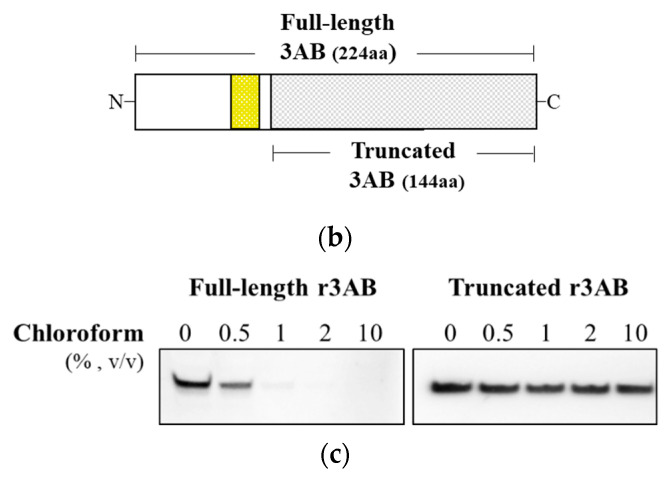
Identification of 3AB protein regions affected by chloroform. (**a**) The topology of 3AB was estimated by the TMHMM program. The *x*-axis indicates the amino acid sequence position, and the *y*-axis shows the possibility of regions located in intracellular (blue line), extracellular (pink line), and transmembrane (red line and stripe) spaces. (**b**) The full-length and truncated 3AB amino acid regions for cloning into the pET28a vector were indicated. The yellow box represents a transmembrane domain. (**c**) Purified recombinant full-length and truncated 3AB proteins were treated with various concentrations of chloroform, and then, 3AB protein was detected by Western blot using anti-FMDV 3B monoclonal antibody.

To define the region of 3AB that causes NSP removal by chloroform, the characteristics of the amino acid residues that make up 3AB were analyzed. The 18 amino acid residues (59th–76th) at the N-terminus of 3AB were identified as transmembrane domains by the TMHMM program (Figure 2a). To investigate whether the transmembrane domain is affected by chloroform, full-length 3AB (224 amino acid residues) and truncated 3AB (144 amino acid residues), in which the N-terminal 80 amino acid residues were deleted, were designed (Figure 2b). Although the full-length 3AB disappeared in a concentration-dependent manner with chloroform treatment, the truncated 3AB was not affected, and the protein remained constant even at a concentration of 10% (*v*/*v*) chloroform (Figure 2c).

### 3.3. Scale-Up Production of FMD Vaccine Antigen by Applying Chloroform Treatment Process

**Figure 3:**
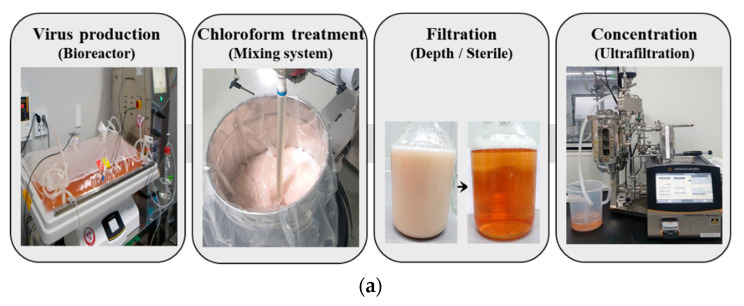

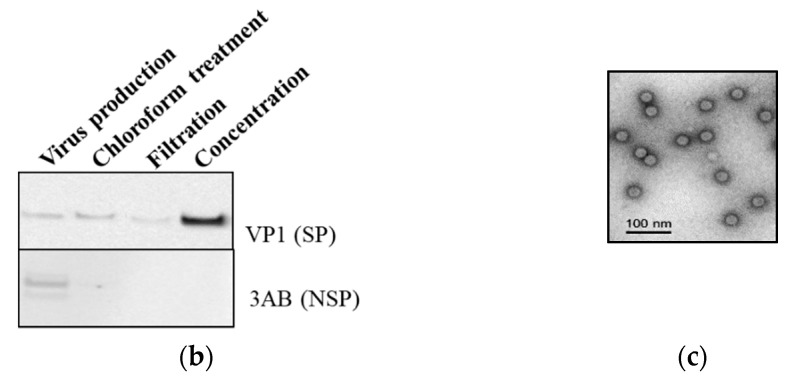
Production of large-scale FMD vaccine antigen using chloroform treatment process. (**a**) The detailed procedure for manufacturing FMD vaccine antigen is shown in images. Description of images from left to right: (i) The O/Boeun/SKR/2017 virus was produced using a wave-type bioreactor. (ii) The 20 L of virus culture supernatant was treated with 2% (*v*/*v*) of chloroform and mixed with an electronic overhead stirrer. (iii) The supernatant was filtrated in the order of depth and sterile filters. (iv) The filtrate was concentrated 10-fold by ultrafiltration. (**b**) FMDV SP (VP1) and NSP (3AB) were detected by Western blot analysis using anti-FMDV VP1 and 3B monoclonal antibodies. (**c**) The 10-fold concentrated vaccine antigens were negatively stained and analyzed for morphology by a transmission electron microscope.

The scale-up of the FMD vaccine antigen with chloroform treatment was verified at a volume of 20 L (Figure 3a). The FMDV culture supernatant was produced in a single-use wave bioreactor and mixed with 2% (*v*/*v*) chloroform using a mechanical mixer. After mixing with chloroform, the opaque solution was transformed into a transparent solution by depth filtration. After sterile filtration, the clarified FMD culture supernatant was concentrated 10-fold using an ultrafiltration device. To investigate the effect of chloroform on the removal of 3AB protein from the FMDV culture supernatant, Western blotting was performed using antibodies against VP1 (SP) and 3B (NSP). While VP1 was detected throughout the process, 3AB was not detected, even in the ten-fold concentrate after chloroform treatment (Figure 3b). Depth filtration was sufficient to remove NSPs as an alternative to centrifugation after chloroform treatment. We examined the integrity of the FMDV particles by electron microscopy, and particles with a diameter of 25–30 nm were observed in the final vaccine antigen concentrate (Figure 3c).

## 4. Discussion

Among the various types of FMDV production media tested in our laboratory, Cellvento was selected for convenience of use [15]. Therefore, we previously evaluated the 3AB removal effect of chloroform in Cellvento medium only [11]. Because there were differences in the components of each medium, we applied ProVero and DMEM to investigate whether chloroform treatment had the same effect on other media. The reason that chloroform did not exert a 3AB removal effect in DMEM in this study might be attributed to certain components. However, because only part of the ingredients of the media are known, it is difficult to determine which component is attributable to this discrepancy. Instead, we found clues that inhibited the effects of chloroform in buffers of defined compositions. In the case of RIPA buffer, which is widely used for intracellular protein analysis after transfection of plasmid DNA into mammalian cell lines [19,20], 3AB protein was not removed by chloroform treatment. In contrast, the chloroform effect was observed in Tris-HCl buffer, suggesting that the detergent contained in the RIPA buffer affected the 3AB removal effect of chloroform. It was speculated that the effect of chloroform on the hydrophobic region of the protein was hindered by the detergent, which increased the solubility of the protein.

The FMDV genome encodes eight different NSPs (L, 2A, 2B, 2C, 3A, 3B, 3C, and 3D) [21]. Although various hydrophobic regions are observed among them [22], amino acid residues 59–76 of the 3A protein are predicted to have transmembrane domain properties. Based on this, we hypothesized that the highly hydrophobic domain of 3AB was removed from the aqueous layer because it dissolved in the chloroform layer. Similar results have been reported, in which most of the identified chloroform-soluble membrane proteins are highly hydrophobic proteins containing several putative membrane-spanning domains [23]. In a previous study, the recombinant 3AB protein, in which 80 amino acid residues were truncated at the N-terminus of 3AB, was reported to be soluble in *E. coli* without forming inclusion bodies [24]. As expected, the soluble recombinant 3AB protein with a truncated transmembrane domain was unaffected by chloroform. A previous study on the membrane topology of 3A protein [25] also supports our interpretation of the chloroform effect. According to the model, the 3A protein interacts with the endoplasmic reticulum membrane through its hydrophobic region, while its N- and C-termini face the cytosol. In addition, non-ionic detergent conditions and deletion of the N-terminus of 3A significantly increased its solubility.

Initially, for a scale-up study of the chloroform treatment process, the effect of chloroform on 3AB in a bench scale 2-L bioreactor was investigated. The chloroform effect was closely related to the shearing force of the impeller attached to the bioreactor. As the volume of the virus culture supernatant in the vessel increased, it was necessary to increase the chloroform concentration or impeller agitation speed for the 3AB removal effect (Figure S1). When the volume of the virus culture supernatant in the vessel was 0.6 L (minimum working volume of the 2 L bioreactor), 3AB was effectively removed when mixed with 2% chloroform at 750 rpm. However, when the volume was increased to 2 L (maximum capacity), stirring at 2000 rpm was required to remove 3AB at 2% (*v*/*v*) chloroform treatment, whereas, at 10% (*v*/*v*) chloroform treatment, to exert the same effect, stirring at 750 rpm was required.

Based on these results, the chloroform treatment process was applied to 20 L of virus culture supernatant, and 2% (*v*/*v*) chloroform was mixed with a single-use modular mixing system; as a result, 3AB was effectively removed by mixing at 350 rpm. Depth filtration, as a subsequent chloroform treatment process, significantly removed chloroform and greatly improved the turbidity of the virus culture supernatant without centrifugation. After performing sterile filtration, it was confirmed that the filter membrane was not affected by chloroform, and its integrity was maintained. Therefore, chloroform at a concentration of 2% (*v*/*v*) is considered suitable for filtration processes.

Ultrafiltration alone cannot remove 3AB protein [11], but since 3AB was eliminated by mixing with chloroform and depth filtration in the previous step, ultrafiltration concentration could be performed as a final step without concern for NSP remaining in the FMD vaccine antigens.

The application of chloroform during vaccine production may raise concerns regarding the safety of target animals. Therefore, we verified the safety of the FMD vaccine applied with the chloroform treatment process according to National Standards for veterinary biologics (Evaluation criteria: There should be no (i) hypersensitivity reaction within 2 h after vaccination, (ii) side effects such as suppuration, necrosis, and fever at the injection site for 14 days, and (iii) clinical symptoms of FMD during the observation period) in three pigs (Figure S2).

## 5. Conclusions

We found that the effect of chloroform on the removal of 3AB is due to the properties of the transmembrane domain and a downstream process that can produce high-purity FMD vaccine antigen by applying chloroform treatment was proposed. This novel process is expected to simplify the conventional process for manufacturing FMD vaccine antigens, and ultimately contribute to reducing the cost of vaccine production.

## Data Availability

Not applicable.

## Supplementary Materials

The following supporting information can be downloaded at: https://www.mdpi.com/article/10.3390/vaccines10071018/s1, Figure S1: Optimization of chloroform treatment parameters at a bench scale 2-L bioreactor. Figure S2. Safety evaluation of FMD vaccine produced by chloroform treatment.
