# Supplementary material for: Factors Involved in Removing the Non-Structural Protein of Foot-and-Mouth Disease Virus by Chloroform and Scale-Up Production of High-Purity Vaccine Antigens
Source: Vaccines (Basel). 2022 Jun 24;10(7):1018. doi: 10.3390/vaccines10071018 (PMC9319003; doi:10.3390/vaccines10071018)
Supplement: Supplementary file 1 [file vaccines-10-01018-s001.zip › Figure S1.pdf]

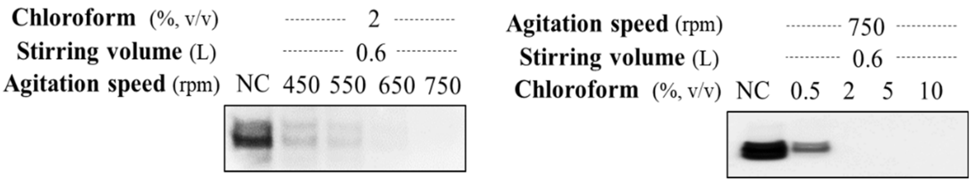

(a)

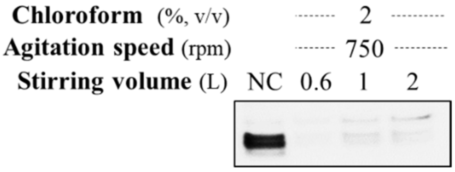

(b)

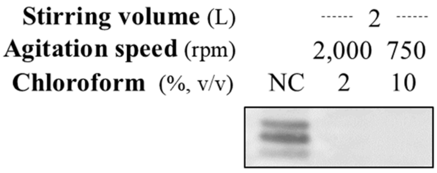

(c)

Figure S1. Optimization of chloroform treatment parameters at a bench scale 2-L bioreactor. (a) Chloroform at 2% (v/v) was added to 0.6 L of the O/Boeun/SKR/2017 virus culture supernatant and mixed at 450, 550, 650, and 750 rpm. (b) Chloroform at 0, 0.5, 2, 5, and 10% (v/v) was added and mixed at 750 rpm. (c) Chloroform at 2% (v/v) was added to 0.6, 1, and 2 L of the virus culture supernatant and mixed at 750 rpm. (d) Two liters of virus culture supernatant was treated with 2% and 10% (v/v) chloroform and mixed at agitation speeds of 2,000 rpm and 750 rpm, respectively. The agitation time for chloroform mixing was 30 min. The 3AB was detected by western blot analysis using anti-FMDV 3B monoclonal antibody.
