# Supplementary material for: Factors Involved in Removing the Non-Structural Protein of Foot-and-Mouth Disease Virus by Chloroform and Scale-Up Production of High-Purity Vaccine Antigens
Source: Vaccines (Basel). 2022 Jun 24;10(7):1018. doi: 10.3390/vaccines10071018 (PMC9319003; doi:10.3390/vaccines10071018)
Supplement: Supplementary file 1 [file vaccines-10-01018-s001.zip › Figure S2.pdf]

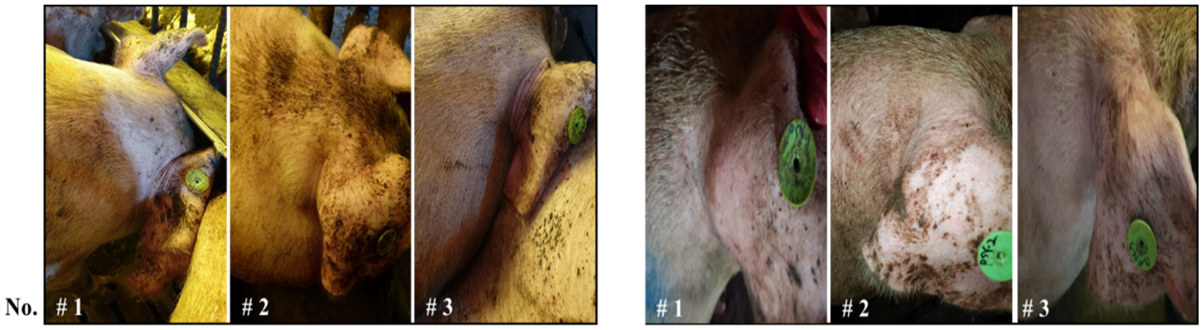

(a) (b)

Figure S2. Safety evaluation of FMD vaccine produced by chloroform treatment. To prepare a FMD vaccine, inactivated FMDV type O Boeun virus culture supernatant was mixed with 10% (v/v) chloroform by inverting for 5 min, and centrifuged at 3,000× g for 15 min. The aqueous layer on the top of the organic solvent layer was harvested and concentrated to a final concentration of 15 μg/mL by an ultrafiltration device. Saponin (Sigma-Aldrich) and aluminum hydroxide gel (General Chemical, NJ, USA) were added to antigens, and then the ISA 206 VG adjuvant (Seppic, Paris, France) was added in a ratio of 1:1. Clinical signs, hypersensitivity, and side effects were observed for 2 weeks after immunization of three pigs at the ear-root-neck area with FMD vaccine in a double dose (30 μg/ 2 mL). (a) 2 hr after vaccination, (b) 2 weeks after vaccination.
